# Radiation Induced Upregulation of DNA Sensing Pathways is Cell-Type Dependent and Can Mediate the Off-Target Effects

**DOI:** 10.3390/cancers12113365

**Published:** 2020-11-13

**Authors:** Tanja Jesenko, Masa Bosnjak, Bostjan Markelc, Gregor Sersa, Katarina Znidar, Loree Heller, Maja Cemazar

**Affiliations:** 1 Department of Experimental Oncology, Institute of Oncology Ljubljana, Zaloska cesta 2, 1000 Ljubljana, Slovenia; tjesenko@onko-i.si (T.J.); mbosnjak@onko-i.si (M.B.); bmarkelc@onko-i.si (B.M.); gsersa@onko-i.si (G.S.); kznidar@onko-i.si (K.Z.); 2 Faculty of Medicine, University of Ljubljana, Vrazov trg 2, 1000 Ljubljana, Slovenia; 3 Faculty of Health Sciences, University of Ljubljana, Zdravstvena pot 5, 1000 Ljubljana, Slovenia; 4 Department of Medical Engineering, University of South Florida, 4202 E Fowler Avenue, Tampa, FL 33620, USA; lheller@usf.edu; 5 Faculty of Health Sciences, University of Primorska, Polje 42, 6310 Izola, Slovenia

**Keywords:** DNA sensors, irradiation, bystander effect, abscopal effects, immunostimulation

## Abstract

**Simple Summary:**

Radiotherapy was shown to act as in situ vaccination, which can mediate systemic response to cancer in conjunction with immune checkpoint inhibitors. We aimed to investigate the DNA sensing pathways as a fundamental mechanism of immunostimulation by DNA after tumor irradiation. Key distinct DNA sensing pathways were identified in two different cell types abundant in the microenvironment of solid tumors, tumor cells and macrophages. We evaluated dose- and time-dependent expression of DNA sensors and cytokines and assayed for radiation-induced bystander effects. Understanding the timing of the activation of these pathways and subsequent cytokine expression induced by different irradiation doses could improve the treatment schemes for combining immune checkpoint inhibitors or other immunotherapies with the radiation therapy in order to improve the therapy of cancer. Therefore, this paper could complement and provide basis for current clinical studies investigating radio-immunotherapy schedules.

**Abstract:**

Irradiation of tumors generates danger signals and inflammatory cytokines that promote the off-target bystander and abscopal effects, evident especially when radiotherapy is administered in combination with the immune checkpoint inhibitors (ICI). The underlying mechanisms are not fully understood; however, cGAS-STING pathway was recognized as the main mediator. In our study, we demonstrate by immunofluorescent staining that tumor cells as well as macrophages, cell types abundant in the tumor microenvironmeent (TME) accumulate DNA in their cytosol soon after irradiation. This accumulation activated several distinct DNA sensing pathways, most prominently activated DNA sensors being DDX60, DAI, and p204 in tumor cells and DDX60, DAI, p204, and RIG-I in macrophages as determined by PCR and immunofluorescence imaging studies. This was accompanied by increased expression of cytokines evaluated by flow cytometry, TNFα, and IFNβ in tumor cells and IL1β and IFNβ in macrophages, which can alter the TME and mediate off-target effects (bystander or abscopal effects). These results give insight into the mechanisms involved in the stimulation of antitumor immunity by radiation.

## 1. Introduction

For several decades, the central dogma in the field of radiotherapy was that radiation effects are restricted only to the irradiated cells, as a direct consequence of unrepaired or misrepaired DNA damage. First “out of field” or off-target effects in humans were reported back in the fifties and were defined as “action at a distance from the irradiated volume but within the same organism” [1]. Almost 40 years later, in 1992, Nagasawa and Little [2] observed increased cell damage in nonirradiated cells neighboring the cells irradiated by low-dose alpha particles. The finding was contrary to common knowledge at that time, but research later on demonstrated that the effect was mediated by factors released from irradiated cells and can cause telomere aberrations, DNA damage, and consequently cell death in nonirradiated cells. The phenomenon was defined as radiation-induced bystander effect (RIBE), which now refers to the plethora of biological phenomena, occurring in nonirradiated cells because of signal transmission from irradiated cells [3]. RIBE can be mediated by soluble factors, such as cytokines or reactive oxygen species (ROS) [3], which can be released from the cells or can be transported through gap junctions [4], or packed into extracellular vesicles in order to travel to the neighboring cells [5]. In neighboring cells, RIBE was demonstrated to be connected to several hallmarks of cancer and can act like a double-edged sword mediating antitumor or protumor responses [3].

As radiotherapy is a mainstay of cancer treatment with approximately one half of all cancer patients receiving it as part of their standard of care, RIBE is being increasingly investigated mainly due to its potentiation of the local tumor response and its possible involvement in normal tissue damage and the risk of secondary cancers caused by radiation exposure [6]. Since the era of immune checkpoint inhibitors (ICI), another “off-target” phenomenon termed “abscopal effect” of radiotherapy is gaining attention. Radiotherapy, mostly when delivered in high doses per fraction such as in stereotactic body radiation therapy (SBRT) and stereotactic radiosurgery (SRS), was shown to act as in situ vaccination, which can mediate systemic response to cancer in conjunction with ICIs [7,8]. The mechanisms of radiation-induced abscopal effect remain unclear; however, the immune system is recognized as a primary mediator [9]. In the tumors, changes induced by ionizing radiation generate damage-associated molecular patterns (DAMPs) and inflammatory cytokines that promote the ability of dendritic cells to cross-represent released tumor antigens to T cells [10]. The mechanistic insight into this phenomenon was published in 2017, demonstrating that lower doses of radiation induced the accumulation of double stranded DNA (dsDNA) in the cytosol of tumor cells, which in turn activated the DNA sensor cyclic GMP-AMP synthase (cGAS) and its downstream adaptor stimulator of interferon genes (STING). This results in type I interferon (IFN) induction, required to recruit and activate Batf3-dependent dendritic cells into the tumor [11]. The latter are essential for priming of tumor-specific CD8 T cells that, in the presence of ICI, mediate complete and durable regression of the irradiated and nonirradiated tumors (abscopal effect) [11].

Besides cGAS-STING pathway, multiple distinct DNA sensing pathways were identified in different types of cells over the last two decades, which can be activated in response to the presence of nucleic acids in the cytosol of cells [12]. Proteins that sense DNA i.e., DNA sensors can be endosomic or cytosolic [7]. Their ligands and signaling cascades are not completely characterized; however, cytosolic DNA sensors’ binding is known to control the production of proinflammatory cytokines and interferons. DNA-dependent activator of interferon regulator factor (DAI/ZBP1/DLM), was the first cytosolic DNA sensor described in 2007 [13]. Subsequently, several different DNA sensors were identified including Pyrin and HIN domain (PYHIN) family members absence in melanoma 2 (AIM2) [14] and AIM2- like protein Interferon Gamma Inducible Protein 16 (IFI16) (mouse ortholog p204) [15], nucleases such as three prime repair exonuclease 1 (TREX1) and double-strand break repair protein MRE11 [7], DexD/H-box helicases family DExD/H-Box Helicase 60 (DDX60), retinoic acid-inducible gene I/ DExD/H-Box Helicase 58 (RIG-I/DDX58), DExH-Box Helicase 9 (DHX9), DExH-Box Helicase 36 (DHX36), DExH-Box Helicase 41 (DDX41), DExH-Box Helicase 36 (DDX36) and others [7,16,17,18], and other types of proteins such as enzyme cGAS [19]. The search for cytosolic DNA sensors led to the discovery of Stimulator of IFN genes (STING), also known as MITA, ERIS, MPYS, TMEM173, an adaptor protein located on the endoplasmic reticulum membrane, which after binding to specific DNA sensors, induces the production of Interferon beta (IFNβ) and tumor necrosis factor alpha (TNFα) [20,21]. The activation of DNA sensing pathways can also lead to the induction of several types of cell death [12].

The key distinct DNA sensing pathways responsible for the observed radiation-induced activation of STING pathway [11,22] and the subsequent expression of different cytokines after irradiation is still unknown. Moreover, the expression of cytokines may be cell type dependent and may therefore mediate diverse responses to radiation in the TME, thus contributing to the off-target bystander or abscopal effect. Therefore, the aim of this study was to evaluate the upregulation or activation of distinct DNA sensing pathways after irradiation in two different cell types abundant in the microenvironment of solid tumors. Tumor cells and macrophages were studied, as macrophages are often the most abundant of tumor-resident immune cells and are highly sensitive to irradiation. The respective contributions of these cell types to radiation-induced off-target effects are unknown [23]. For this purpose, we evaluated the dose- and time-dependent upregulation of several DNA sensors, as well as the subsequent activation of the downstream adaptor STING and the transcription factors IRF3, IRF7, and NF-kB in both cell types in cells that survived after irradiation. We evaluated the resulting time-dependent expression of different cytokines and assayed for radiation-induced bystander effect. Furthermore, we evaluated if DNA was released from the irradiated cells into the cell-conditioned medium and whether it was transferred to neighboring (bystander) cells, potentially resulting in a cascade of activation of DNA sensing pathways, which could augment the RIBE.

## 2. Results

### 2.1. Upregulation of DNA Sensing Pathways After Irradiation is Cell TYPE Specific

**Figure 1:**
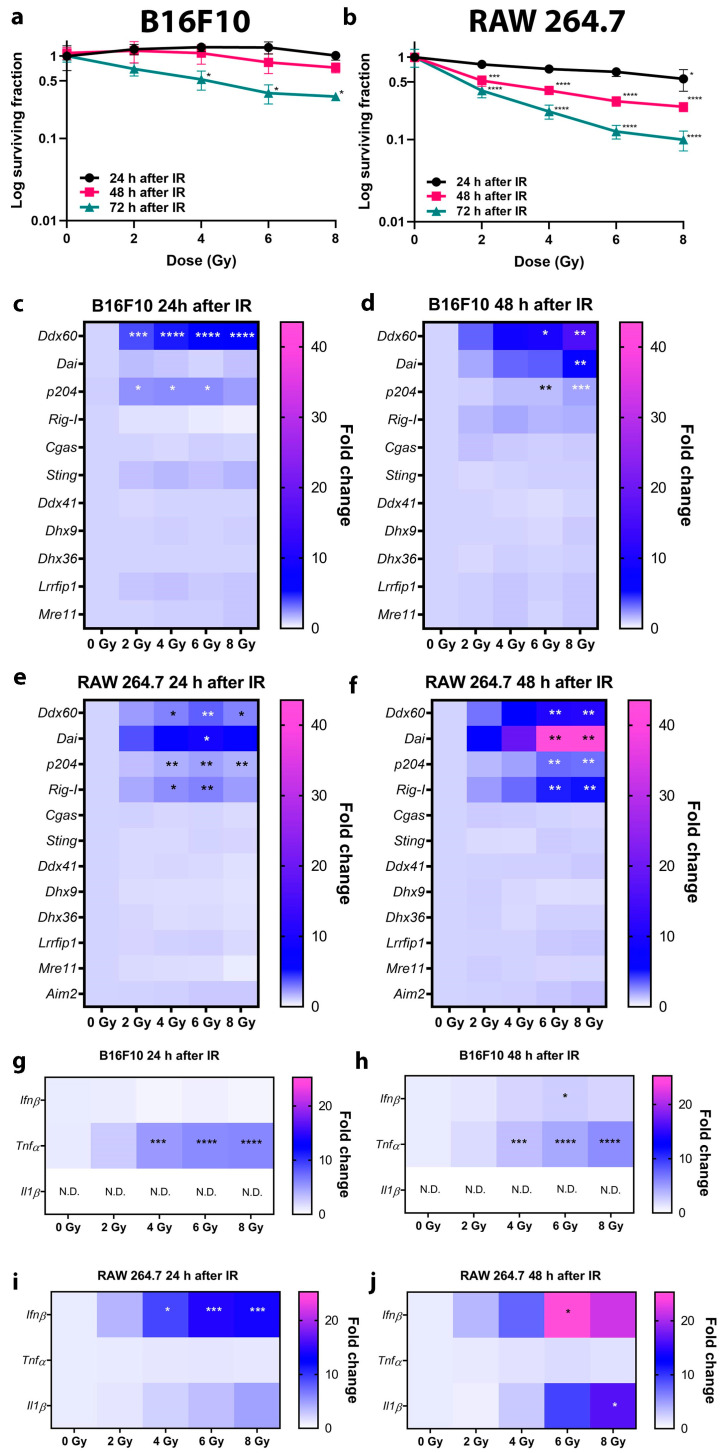
Effect of irradiation on cell survival and upregulation of DNA sensor and cytokine mRNA at different doses of irradiation in early time points in B16F10 tumor cells and RAW 264.7 macrophages. (**a**) Survival of B16F10 cells 24, 48, and 72 h after irradiation with 2, 4, 6, and 8 Gy. *n* = 2–3. Note that the y-axis representing survival fraction is logarithmic. (**b**) Survival of RAW 264.7 cells 24, 48, and 72 h after irradiation with 2, 4, 6, and 8 Gy. *n* = 3. Note that the y-axis representing survival fraction is logarithmic. (**c**) Expression heat maps of DNA sensors 24 h after irradiation of B16F10 cells with 2, 4, 6, and 8 Gy. *n* = 3 and (**d**) expression heat maps of DNA sensors 48 h after irradiation of B16F10 cells with 2, 4, 6, and 8 Gy. *n* = 3. (**e**) Expression heat maps of DNA sensors 24 h after irradiation of RAW 264.7 cells with 2, 4, 6, and 8 Gy. *n* = 3. (**f**) Expression heat maps of DNA sensors 48 h after irradiation of RAW 264.7 cells with 2, 4, 6, and 8 Gy. *n* = 3. (**g**) Cytokine expression heat maps 24 h after irradiation of B16F10 cells with 2, 4, 6, and 8 Gy. *n* = 3. (**h**) Cytokine expression heat maps 48 h after irradiation of B16F10 cells with 2, 4, 6, and 8 Gy. *n* = 3. (**i**) Cytokine expression heat maps 24 h after irradiation of RAW 264.7 cells with 2, 4, 6, and 8 Gy. *n* = 3. (**j**) Cytokine expression heat maps 48 h after irradiation of RAW 264.7 cells with 2, 4, 6, and 8 Gy. *n* = 3. Statistical significance was determined by one-way ANOVA followed by a Dunnett’s multiple comparisons test, *n* = number of biological replicates. * *p* < 0.05, ** *p* < 0.01, *** *p* <0.001, **** *p* < 0.0001 vs. 0 Gy. Non determined (N.D.): Ct value above 40. Cycle; IR: irradiation. Presentation of heat map data in the form of bar graphs can be found in supplementary files (Figure S1).

The murine B16F10 melanoma cell line (further referred as tumor cells) and RAW 264.7 macrophages (further referred as macrophages) were used in this study. To determine the events before the irradiation-induced cell death, cell survival in the first 3 days after irradiation was determined. Cell survival was significantly reduced only at 72 hours (h) after irradiation with 4, 6, and 8 gray (Gy) in the tumor cells (Figure 1a) and at 48 and 72 h at all doses used in the macrophages (Figure 1b). Therefore, 24 and 48 h time points were selected for the rest of the experiments in order to evaluate the effects that take place in irradiated but still viable tumor cells. The same dose was also used for macrophages.

Different DNA sensor mRNAs were upregulated after irradiation and the level of upregulation varied with time and the delivered radiation dose. In general, mRNA expression increased with increasing doses of irradiation and with time after irradiation. At 24 h after irradiation, the expression of DNA sensor *Ddx60* and *p204* mRNAs was significantly upregulated in tumor cells (Figure 1c). At 48 h after irradiation, the expression of *Ddx60* and *p204* was maintained while additionally *Dai* mRNA became upregulated at the highest dose in tumor cells (Figure 1d). The other tested DNA sensors (*Rig-I*, *Cgas*, *Sting*, *Ddx41*, *Dhx9*, *Dhx36*, *Lrrfip1*, *Mre11*) were expressed in the tumor cells but were not significantly upregulated after irradiation. In macrophages, *Ddx60*, *Dai*, *p204,* and *Rig-I* mRNAs were significantly upregulated 24 and 48 h after irradiation (Figure 1e,f). Other tested DNA sensors (*Cgas*, *Sting*, *Ddx41*, *Dhx9*, *Dhx36*, *Lrrfip1*, *Mre11,* and additionally *Aim2,* which is absent in melanoma cells) were expressed in macrophages but were not significantly upregulated after irradiation. In the case of the same upregulated DNA sensors, the upregulation was more pronounced in the macrophages as in the tumor cells, which was significant for *Dai.*

The upregulation of cytokine mRNAs can correspond to the upregulation of DNA sensor mRNAs. In tumor cells, the upregulation of *Tnfα* expression was dependent on the irradiation dose both 24 and 48 h after irradiation (Figure 1g,h). The expression of *Ifnβ* significantly increased 48 h after irradiation (Figure 1h). These cells did not express *Il1β* in control cells or at any of the tested doses (Figure 1g,h). In contrast to the tumor cells, irradiation did not upregulate the expression of *Tnfα* in macrophages. The upregulation of *Ifnβ* expression was dependent on the irradiation dose and was significantly upregulated after both 24 and 48 h (Figure 1i,j). Further, the expression of *Il1β* was also significantly increased at 48 h after irradiation. (Figure 1j). In macrophages, *Ifnβ* mRNA expression was significantly increased compared to tumor cells.

To summarize, the upregulation of DNA sensors and cytokines after irradiation differed between the tumor cells and macrophages. Distinct DNA sensors were upregulated, with the most prominent being *Ddx60*, *Dai* and *p204* in tumor cells and *Ddx60*, *Dai*, *p204*, and *Rig-I* in macrophages. The upregulation was accompanied by upregulation of distinct cytokines with the most prominent being *Tnfα* and *Ifnβ* in tumor cells and *Ifnβ* and *Il1β* in macrophages. The level of upregulation increased with the delivered dose and time. However, only the increase in *Dai* and *Ifnβ* mRNA expression was significant between 24 and 48 h in tumor cells and macrophages. Overall, the upregulation of DNA sensors and cytokine mRNAs was more pronounced in the macrophages compared to tumor cells, which was significant in the case of *Dai* and *Ifnβ*.

### 2.2. Irradiation Induced Cytosolic Accumulation of Micronuclei and dsDNA and Translocation of Proteins Involved in DNA Sensing Pathways

The accumulation of micronuclei and dsDNA in the cell cytosol as a source of cytosolic DNA and translocations of proteins involved in DNA sensing pathways were evaluated for further elaboration of increased expression of DNA sensors post irradiation of tumor cells or macrophages. For proof-of-principle imaging studies, irradiation dose of 6 Gy was selected, which is used in schedules for SBRT and where a significant upregulation of DNA sensors and cytokines in tumor cells and macrophages was observed (Figure 1).

**Figure 2:**
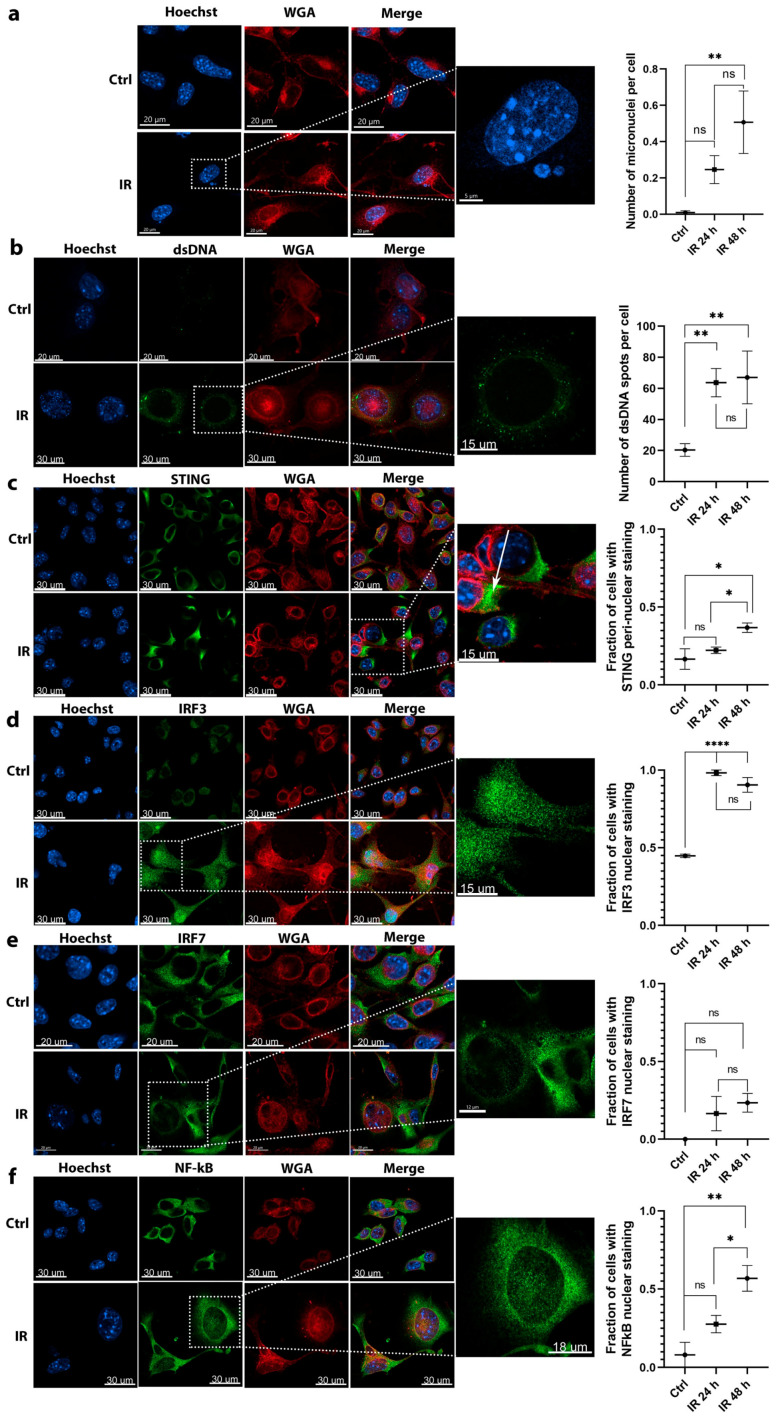
Imaging studies revealed the activation of molecules involved in DNA sensing pathways in response to 6 Gy irradiation of B16F10 tumor cells. (**a**) The accumulation of micronuclei in cell cytosol after irradiation; scale bar = 20 μm (Ctrl, IR), 5 μm (inset); *n* = 13–15. (**b**) Irradiation-dependent accumulation of dsDNA in the cytosol of cells. Arrows are pointed to representative dsDNA spots; scale bar = 20 μm (Ctrl), 30 μm (IR), 15 μm (inset); *n* = 12–16. (**c**) Irradiation induced translocation of STING from cytoplasm to peri-nuclear punctate structures (arrows); scale bar = 30 μm (Ctrl, IR), 15 μm (inset); *n* = 5. (**d**) Irradiation induced translocation of IRF3 transcription factor from the cytoplasm to the nucleus; scale bar = 30 μm (Ctrl, IR), 15 μm (inset); *n* = 5. (**e**) Irradiation failed to induce a significant translocation of IRF7 transcription factor from the cytoplasm to the nucleus; scale bar = 20 μm (Ctrl, IR), 12 μm (inset); *n* = 9. (**f**) Irradiation induced translocation of NF-κB transcription factor from the cytoplasm to the nucleus; scale bar = 30 μm (Ctrl, IR), 18 μm (inset); *n* = 12 cells. Statistical significance was determined by one-way ANOVA followed by a Dunnett’s multiple comparisons test. * *p* < 0.05, ** *p* < 0.01, **** *p* < 0.0001 vs. Ctrl group; *n* = number of fields of view. Images of control nonirradiated cells (denoted by Ctrl), and irradiated cells 48 h after 6 Gy irradiation (denoted by IR) in all imaged channels are presented. In the graphs, *x* axis label Ctrl represents quantified values of control (nonirradiated) cells and labels IR 24 h and IR 48 h represent quantified values of 6 Gy irradiated cells 24 and 48 h after irradiation, respectively. ns = not significant.

After irradiation, the accumulation of micronuclei (Figure 2a) and dsDNA (Figure 2b) was detected in the cytosol of tumor cells. The number of micronuclei significantly increased 48 h after irradiation (Figure 2a). A significant 3-fold increase in the number of dsDNA spots were detected at 24 and 48 h after irradiation (Figure 2b) (IR 24 h and IR 48 h groups). Interestingly, approximately 20 dsDNA spots were present per cell in control nonirradiated cells (Ctrl group), indicating that dsDNA is already present in the cytosol of tumor cells, possibly interfering with the normal response to cytosolic DNA.

The activation of STING, which interconnects multiple upstream DNA sensing pathways, was evaluated by its translocation to peri-nuclear punctate structures, which can represent the Golgi apparatus, peri-nuclear vesicles, and autophagy-related compartments. In control, nonirradiated tumor cells (Ctrl group), STING staining was dispersed throughout the cytoplasm. The fraction of cells with peri-nuclear STING staining tended to increase 24 h after irradiation and significantly increased by 2-fold 48 h after IR (Figure 2c).

The translocation of downstream transcription factors known to induce the expression of cytokines as the result of DNA sensing were then assessed. The activation of transcription factors IRF3 and NFκB was determined through their translocation from the cytoplasm to the nucleus. In irradiated groups, an approximately 2-fold increase in the number of tumor cells with IRF3 nuclear staining was detected compared to control nonirradiated cells (Ctrl group). The nuclear translocation of IRF3 significantly increased 24 and 48 h after irradiation (Figure 2d) compared to control cells, however there was no significant difference between the 24 and 48 h group. The IRF7 translocation tended to increase 24 and 48 h after irradiation (IR 24 and 48 h groups) (Figure 2e), however was not significant. The translocation of NFκB was evident earlier at 24 h after irradiation, when a nonsignificant 4-fold increase in the number of tumor cells with nuclear staining was detected in the irradiated cells (IR 24 h group). This further increased at 48 h after irradiation (IR 48 h group), when statistically significant 8-fold more cells had NFκB nuclear staining compared to control nonirradiated cells (Figure 2f).

Contrary to tumor cells, the proof-of-principle imaging studies in macrophages could be performed only 24 h after irradiation due to the evident cell death 48 h after irradiation (Figure 1b).

**Figure 3:**
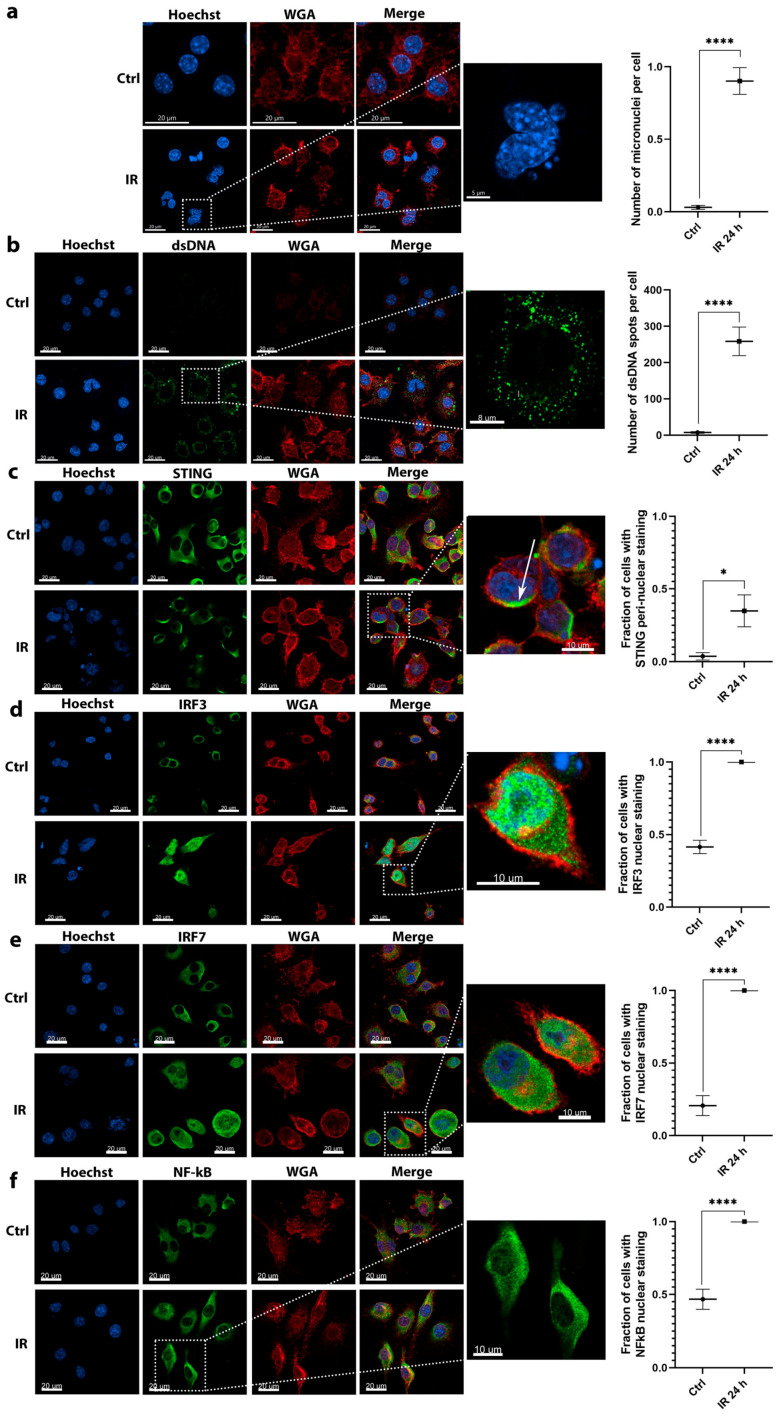
Imaging studies revealed the activation of molecules involved in DNA sensing pathways in response to 6 Gy irradiation of RAW 264.7 macrophages. (**a**) The accumulation of micronuclei in cell cytosol after irradiation; scale bar = 20 μm (Ctrl, IR), 5 μm (inset); *n* = 13. (**b**) Irradiation-dependent accumulation of dsDNA in the cytosol of cells; scale bar = 20 μm (Ctrl, IR), 8 μm (inset); *n* = 7–8. (**c**) Irradiation induced the translocation of STING from cytoplasm to peri-nuclear punctate structures (arrows); scale bar = 20 μm (Ctrl, IR), 10 μm (inset); *n* = 7. (**d**) Irradiation induced the translocation of IRF3 transcription factor from the cytoplasm to the nucleus; scale bar = 20 μm (Ctrl, IR), 10 μm (inset); *n* = 7. (**e**) Irradiation induced the translocation of IRF7 transcription factor from the cytoplasm to the nucleus; scale bar = 20 μm (Ctrl, IR), 10 μm (inset); *n* = 6. (**f**) Irradiation induced the translocation of NFκB transcription factor from the cytoplasm to the nucleus; scale bar = 20 μm (Ctrl, IR), 10 μm (inset); *n* = 6. Statistical significance was determined by using unpaired two-tailed Student’s *t*-test. t. * *p* < 0.05, **** *p* < 0.0001; *n* = number of fields of view. Images of control nonirradiated cells (denoted by Ctrl) and 6 Gy irradiated cells at 24 h after irradiation (denoted by IR) in all imaged channels are presented. In the graphs, *x* axis label Ctrl represents quantified values of control (nonirradiated) cells and label IR 24 h represents quantified values of 6 Gy irradiated cells 24 h after irradiation.

Similar to tumor cells, micronuclei (Figure 3a) and dsDNA (Figure 3b) also accumulated in the cytosol of macrophages 24 h after irradiation (IR group). The accumulation was more pronounced as in tumor cells. After irradiation, macrophages presented with deformed lobulated nuclei with numerous micronuclei present in the cytosol (Figure 3a). Evident accumulation of dsDNA was also detected, on average 250 dsDNA spots were detected per one macrophage cell (IR 24 h group) representing a significant 34-fold increase in the number of dsDNA spots after irradiation (Figure 3b). In contrast, irradiated tumor cells contained only around 60 dsDNA spots per cell after irradiation, indicative of a more potent response in macrophages. Furthermore, contrary to tumor cells only a few dsDNA spots were observed in nonirradiated macrophages (Figure 3b, Ctrl group).

The significant activation of STING was also detected in macrophages with the fraction of cells with peri-nuclear STING staining increased for approximately 7-fold at 24 h after irradiation (Figure 3c).

Contrary to the tumor cells, the translocation of all three evaluated transcription factors from the cytoplasm to the nucleus was significantly increased at 24 h after irradiation. Specifically, the determined increase in the number of cells with nuclear staining was 2.3-fold for IRF3 (Figure 3d), 4-fold for IRF7 (Figure 3e), and 2-fold for NFκB (Figure 3f).

Taken together, the accumulation of micronuclei and dsDNA in the cytosol was more pronounced in macrophages as in tumor cells, suggesting the source for the more potent upregulation of the DNA sensors and cytokine mRNAs presented in Figure 1. Furthermore, the activation of STING and downstream transcription factors was more potent in macrophages; nearly all detected macrophage cells presented with the nuclear staining for the tested transcription factors.

### 2.3. The Expression of Cytokines Mediated by the Activation of DNA Sensing Pathways is Involved in RIBE

**Figure 4:**
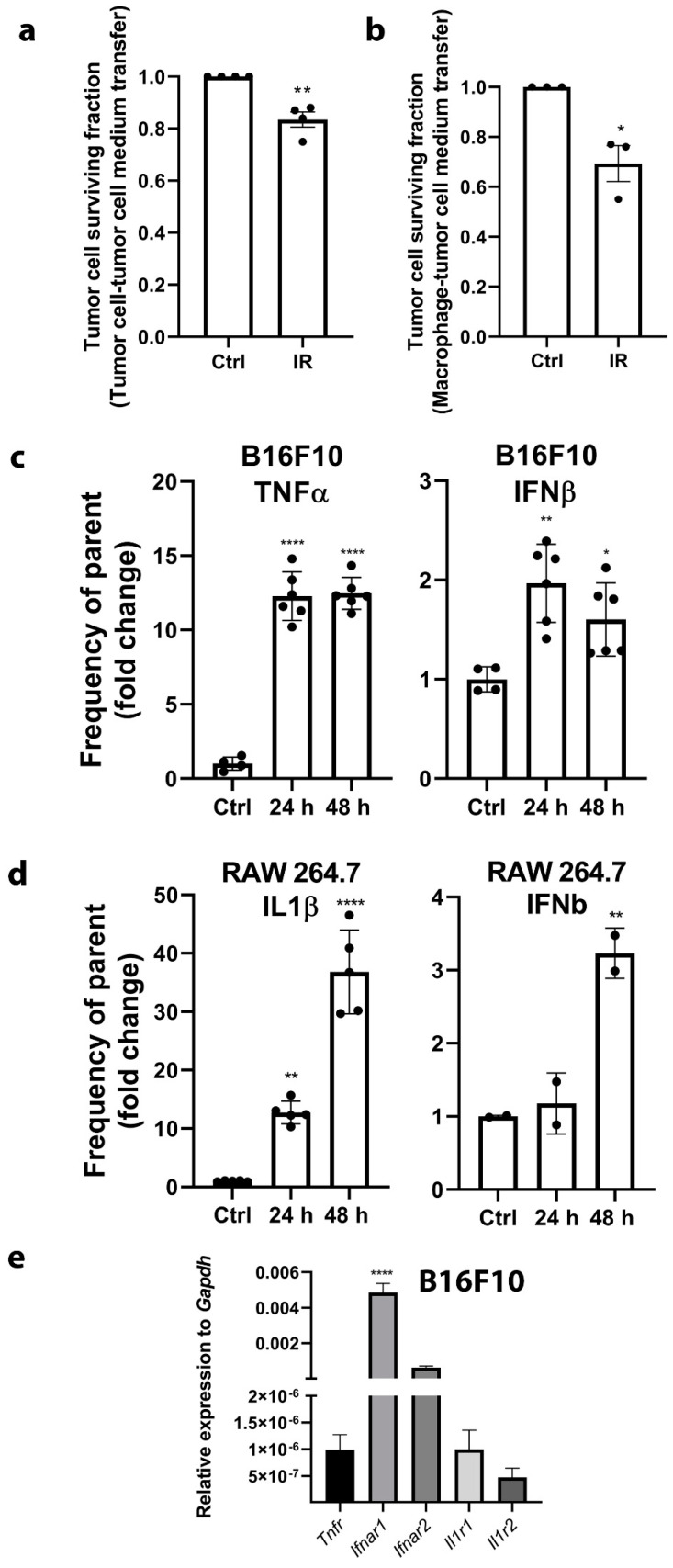
The transfer of cell-conditioned medium from irradiated cells to nonirradiated cells reduced the clonogenic survival of tumor cells (RIBE) and was accompanied by the expression of cytokines mediated by activation of DNA sensing pathways. (**a**) Clonogenic survival of B16F10 tumor cells after the transfer of cell-conditioned medium of control nonirradiated B16F10 cells (Ctrl group) or 6 Gy irradiated B16F10 cells (IR group); *n* = 4. (**b**) Clonogenic survival of B16F10 tumor cells after the transfer of cell-conditioned medium of control nonirradiated RAW 264.7 macrophages (Ctrl group) or 6 Gy irradiated RAW 264.7 macrophages (IR group); *n* = 3. (**c**) The protein expression fold change of TNFα and IFNβ at 24 h (24 h group) and 48 h (48 h group) after irradiation of B16F10 cells normalized to control nonirradiated cells (Ctrl). The expression was evaluated by intracellular flow cytometry and represents the frequency of parent population (TNFα or IFNβ positive subset of single live cells); *n* = 4–6. (**d**) The protein expression fold change of IL1β and IFNβ at 24 h (24 h group) and 48 h (48 h group) after irradiation of RAW 264.7 macrophages normalized to control nonirradiated cells (Ctrl). The expression was evaluated by intracellular flow cytometry and represents the frequency of parent population (IL1β or IFNβ positive subset of single live cells); *n* = 5–6. (**e**) Relative expression of cytokine receptors *Tnfr, Ifnar1, Ifnar2, Il1r1,* and *Il1r2* in B16F10 cells normalized to *Gapdh* expression demonstrating present binding targets for released cytokines; *n* = 6. Statistical significance was determined by one-way ANOVA followed by a Dunnett’s multiple comparisons test for panels A, B, C, D or Tukey’s multiple comparisons test for panel E, *n* = number of biological replicates. * *p* < 0.05, ** *p* < 0.01, **** *p* < 0.0001 vs. Ctrl. *n* = number of biological replicates.

The induction of RIBE was evaluated by clonogenic survival of tumor cells after the transfer of cell-conditioned medium of control nonirradiated cells (Ctrl group) or 6 Gy irradiated cells (IR group) from both cell lines, tumor cells and macrophages. A significant survival reduction of tumor cells was observed after the transfer of cell-conditioned medium from either irradiated tumor cells (Figure 4a) or macrophages (Figure 4b) of approximately 20% or 30%, respectively. There was no significant difference in clonogenic survival between the addition of tumor- or macrophage- cell-conditioned medium. This demonstrated that soluble factors present in the irradiated cell-conditioned medium 24 h post irradiation reduced the survival of bystander nonirradiated cells.

These bystander effects may be mediated by the expression of several upregulated cytokines, which accompanied the activation of DNA sensing pathways (Figure 1). The increased expression of the TNFα and IFNβ proteins was confirmed by intracellular flow cytometry in tumor cells at 24 and 48 h after 6 Gy irradiation. The percent of cytokine producing cells (frequency of parent population) significantly increased approximately 12-fold for TNFα and 2-fold for IFNβ (Figure 4c). Cytokine expression after 6 Gy irradiation was also confirmed in macrophages. The percent of cytokine expressing cells was higher than in tumor cells. The percent of cytokine expressing macrophages was significantly increased by approximately 38-fold for IL1β and 2.5-fold for IFNβ 48 h post irradiation (Figure 4d).

To confirm that all of the expressed cytokines could potentially bind and activate their receptors in tumor cells, the mRNA expression of interferon-α/β receptors *(Ifnar1* and *Ifnar2)*, tumor necrosis factor receptor (*Tnfr*), and interleukin 1 receptor type 1 and 2 (*Il1r1* and *Il1r2*) was evaluated. All of the tested receptors were expressed in tumor cells, the most abundant being *Ifnar1* (Figure 4e).

### 2.4. The DNA Released after Irradiation Enters Nonirradiated Cells but Does Not Trigger DNA Sensing Pathways

**Figure 5:**
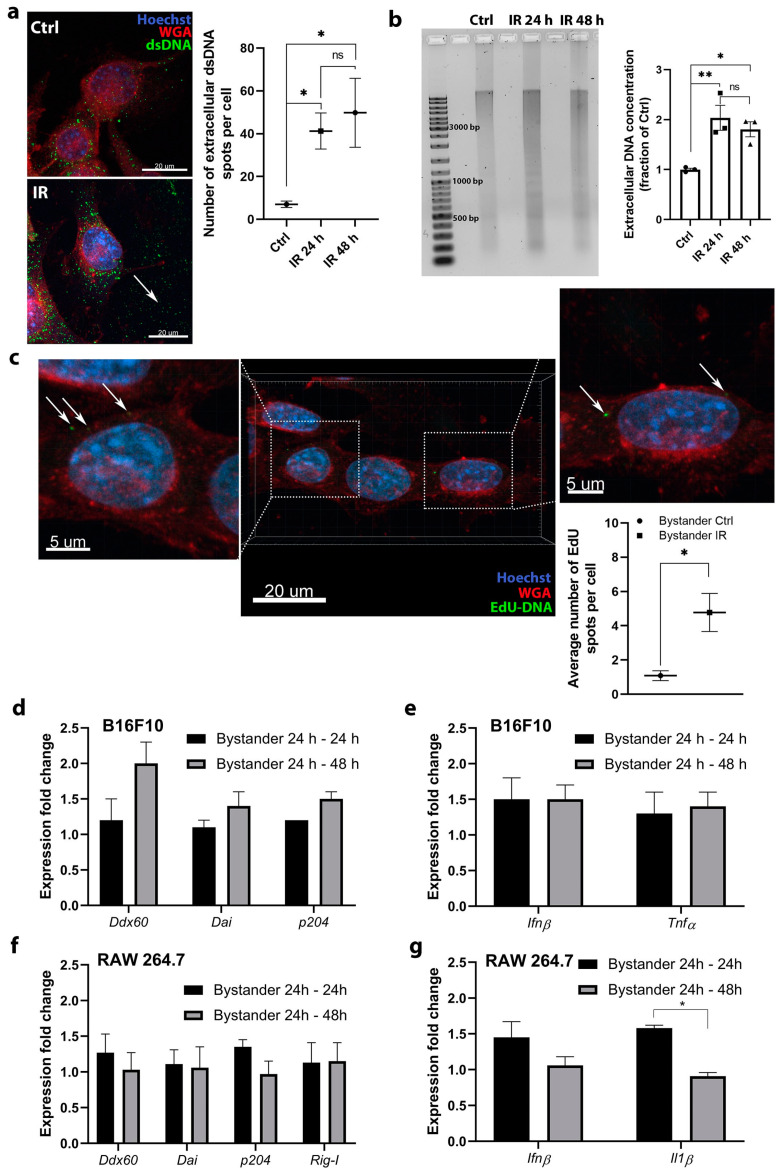
Radiation induced the release of DNA into the cell medium, which entered fresh nonirradiated cells but did not induce the upregulation of DNA sensing pathways and cytokines. (**a**) DNA staining demonstrated that DNA is present outside the irradiated B16F10 cells in a larger amount than control cells; scale bar = 20 μm; *n* = 8–9 (number of quantified images). DNA was detected using anti-dsDNA antibodies; the signal is presented by arrows indicating green spots. Ctrl—control cells, IR—6 Gy irradiated cells. (**b**) Agarose gel analysis and measured concentration of isolated DNA from the cell culture medium from control nonirradiated cells (Ctrl) and after 6 Gy irradiation at 24 h (IR 24 h group) and 48 h (IR 48 h group); *n* = 3. The original image of AGE gel shown in Figure S4. (**c**) The detection of the uptake of DNA with incorporated EdU (arrow) from the cell-conditioned medium of irradiated B16F10 cells into the fresh nonirradiated B16F10 cells. The positive signal on images is presented by arrows indicating green spots and the quantification of images on graph representing average number of EdU spots per cell; scale bar = 20 μm and 5 μm (inset); *n* = 5 (number of quantified images). (**d**) Expression fold change of specific DNA sensors after addition of cell-conditioned medium from 6 Gy irradiated B16F10 cells to fresh nonirradiated B16F10 cells and 24 h incubation (Bystander 24 h–24 h) or 48 h incubation (Bystander 24 h–48 h); *n* = 4–7. (**e**) Expression fold change of specific cytokines after addition of cell-conditioned medium from 6 Gy irradiated B16F10 cells to fresh nonirradiated B16F10 cells and 24 h incubation (Bystander 24 h–24 h) or 48 h incubation (Bystander 24 h–48 h); *n* = 3. (**f**) Expression fold change of specific DNA sensors after addition of cell-conditioned medium from 6 Gy irradiated B16F10 cells to fresh nonirradiated RAW 264.7 macrophages and 24 h incubation (Bystander 24 h–24 h) or 48 h incubation (Bystander 24 h–48 h), *n* = 3. (**g**) Expression fold change of specific cytokines after addition of cell-conditioned medium from 6 Gy irradiated B16F10 cells to fresh nonirradiated RAW 264.7 macrophages and 24 h incubation (Bystander 24 h–24 h) or 48 h incubation (Bystander 24 h–48 h); *n* = 3. For panels (**d**–**g**), the values were normalized to the pertinent control groups i.e., cells to which the cell-conditioned medium of control nonirradiated cells was added. Statistical significance was determined by one-way ANOVA followed by a Dunnett’s multiple comparisons test. * *p* < 0.05, ** *p* < 0.01 vs. Ctrl. *n* = number of biological replicates in (**b**,**d**–**g**).

Another possible mechanism of RIBE was then considered. Specifically, the involvement of DNA sensing pathways in RIBE induced by the uptake of DNA released from the irradiated tumor cells was assessed. The rational for this experiment was to determine if the released DNA can potentiate the RIBE by entering in the bystander cells and enhance the DNA sensing responses. The presence of DNA outside the tumor cells after 6 Gy irradiation was first determined by staining with antibodies against dsDNA (Figure 5a). The quantification of images confirmed that DNA was present outside the irradiated B16F10 cells in a larger amount than control cells (Figure 5a). Then, DNA isolated from cell culture medium was analyzed by gel electrophoresis. Cell-free DNA was highly fragmented with a smeared pattern, which was evident at both time points after irradiation (IR 24 h and IR 48 h group) and also after isolation from the control nonirradiated group of cells (Ctrl group) (Figure 5b). At IR 24 h and IR 48 h, a darker lane was detected indicating a larger quantity of DNA at this time point (Figure 5b). This was confirmed by measuring the concentration of DNA isolated from the cell-conditioned medium, which revealed a significant increase in DNA concentration at 24 and 48 h after irradiation (Figure 5b).

In further experiments, the entering of the released DNA from the cell-conditioned culture medium into the neighboring bystander tumor cells was evaluated to confirm that DNA is able to enter the cell and potentially activate the DNA sensing pathways. Fresh nonirradiated tumor cells were used as a model of neighboring bystander cells, to which the cell-conditioned medium of irradiated cells was added. To ensure that only DNA released from the irradiated tumor cells will be detected, we labeled this DNA by incorporation of 5-ethynyl-2′-deoxyuridine (EdU) prior to irradiation. The transfer of labeled DNA from the cell-conditioned medium of irradiated tumor cells into the fresh tumor cells was then followed by EdU immunofluorescent staining. The transferred EdU labeled DNA in the fresh tumor cells in the form of cytoplasmic spots was detected (Figure 5c), indicating that DNA can indeed enter the cells. However, this event was rare and only a few spots of EdU labeled DNA was observed in the cells, although the number of spots significantly increased compared to the control group (Bystander Ctrl group).

The induction of DNA sensing pathways in the bystander cells by the transferred DNA was evaluated to determine if this process can contribute to or potentiate the RIBE. Only the expression of specific DNA sensors and cytokines that were upregulated after irradiation in each cell type (Figure 1) was evaluated. The transferred cell-conditioned medium of irradiated tumor cells containing DNA failed to significantly induce DNA sensor (Figure 5d) and cytokine (Figure 5e) mRNA upregulation in tumor cells after either 24 h (Bystander 24–24 h) or 48 h (Bystander 24–48 h). 

Similarly, no significant upregulation of DNA sensor (Figure 5f) or cytokine (Figure 5g) mRNA was detected after incubation of the same cell-conditioned medium for 24 h (Bystander 24–24 h) or 48 h (Bystander 24–48 h) in macrophages.

## 3. Discussion

In this study, distinct DNA sensing pathways involved in tumor immune response, modification of TME, and mediation of radiation-induced bystander effects were identified. It was demonstrated that both, tumor cells and macrophages, representing two different cell types abundant in the TME of solid tumors, accumulate DNA in their cytosol soon after high dose irradiation. The accumulated DNA in the cytosol upregulated and activated several distinct DNA sensing pathways, depending on cell type. This resulted in the expression of several cytokines capable of altering the TME [24] and mediating the off-target effects, either by direct killing of neighboring cells or by inducing inflammation and recruitment of immune cells. In addition, it was demonstrated that DNA released by the cells in response to irradiation can also enter the neighboring (bystander) cells, though in very small quantities. However, this DNA was not sufficient to activate the expression of DNA sensing pathways implying that it is also not involved in the potentiation of the radiation-induced bystander effect.

An early response to high dose radiation, when the cells are still viable and the release of DNA is not due to the cell death-induced impaired membrane integrity was the primary objective of this study. The study aimed to compare the early effects in tumor cells and macrophages side-by-side as both cell types are present in the tumors at the time of irradiation and are exposed to the same dose, which is often 6–8 Gy for SBRT fractions or 2–4 Gy for conventional radiotherapy fractions. Before RNA isolation, dead floating cells were carefully removed, and RNA was isolated from the remaining attached cells. Therefore, our results demonstrate the expression profiles of cells that are still viable at that time point.

DNA sensing pathways are complex and intertwined, numerous DNA sensing pathways were identified in different types of cells [7]. The activation of distinct DNA sensors is able to activate the same downstream transcription factors mediating cytokine expression and vice versa, the same DNA sensor repertoire can mediate the induction of different cytokines.

In our study, among several tested DNA sensors, only *Ddx60*, *Dai*, and *p204* were significantly upregulated in tumor cells after irradiation. DDX60 is an interferon inducible cytoplasmic helicase that possesses the ability to bind to RNA and DNA and can mediate INFβ expression through activation of IRF3 or acting as an upstream factor of RIG-I that activates RIG-I signaling [7,18,25]. DAI directly binds DNA through its Z-DNA-binding domains and signal through STING to induce IRF3 and NF-kB activation [7,13]. P204 efficiently binds to DNA motifs and consequently recruits STING [7]. After activation, STING can activate IRF3, IRF7, and NF-κB. These differential responses can mediate the expression of different cytokines, dependent on the cell line and its current status [26]. STING can induce the expression of IFNβ, TNFα, or both [21], the latter seems to be the case in B16F10 tumor cell response to radiation.

A similar but more potent response was observed in macrophages. IFNβ production was higher as in tumor cells and could also be mediated through STING, as in the case of tumor cells. However, STING signaling in macrophages did not result in production of TNFα, demonstrating the differential response in tumor cells and macrophages. The more potent induction of IFNβ and production of IL1β in macrophages were also mediated by the upregulation of one additional DNA sensor *Rig-I*. RIG-I is primarily responsible for the detection of single-stranded and double stranded RNA, generated in the course of a virus infection. However, the work of Chiu et al. demonstrated that cytosolic DNA can be used as a template for RNA polymerase III-driven synthesis of dsRNA, which can bind RIG-I and induce IFNβ production [27]. RIG-I activation can be potentiated by the binding of DDX60, which was also upregulated in our study after irradiation of RAW 264.7 macrophages. RIG-I can mediate the expression of various cytokines through IRF3, IRF7, and NF-κB [7], with IFNβ and IL1β being upregulated after irradiation of RAW 264.7 macrophages in our study. Furthermore, IL1β can be induced also by DNA sensors DAI/ZBP1 and p204 (mouse analog of IFI16) [7,12], which were also significantly upregulated in macrophages following irradiation.

Regarding these data, our proposed mode of action for cellular responses to radiation in terms of DNA sensing pathways for both cell lines is presented in Figure S2. The proposed schemes are based on our current expression and activation data based on qRT-PCR, flow cytometry, and imaging studies of protein translocations. For a more comprehensive scheme, knock-down or knock-out studies would be needed, however, they were not in the scope of this study which is one of the limitations of our study. Our evaluation of DNA sensors was made primarily based on the upregulation of their expression; therefore, we cannot exclude the possibility that the sensors that were not upregulated still bound to the DNA and exerted their function. In our previous study, it was demonstrated that although DHX9 expression was not upregulated in C2C12 myoblasts, the basal level present in the cells was adequate for the detection of plasmid DNA binding to DHX9 as determined by a pull-down assay [28]. Therefore, as irradiation elicits cellular responses that mimic a viral infection through the detection of accumulated cytosolic DNA, there could be patrolling first line cytosolic DNA sensors that immediately bind the DNA, mediating the primary response. These could be evaluated in the future by additional specific tests of interactions or their localization. In this study, a similar effect was observed for STING. Its expression was not upregulated after irradiation of B16F10 cells but its activation by the translocation to the peri-nuclear punctate structures was detected.

In the study of Vanpouille-Box et al. the authors demonstrated the upregulation of Zbp1 (Dai), Ifi204, and Ddx58 (Rig-I) gene expression in TSA tumors 24 h after radiation in vivo with 3 × 8 Gy [11]. These results were confirmed in our study, which also further distinguished the differences in the upregulation between different cell lines, clarifying basic mechanisms of DNA sensing pathways activation by radiation. Contrary to the study of Vanpouille-Box et al. [11], where the upregulation of cGAS-STING pathway on mRNA level was required for type I IFN induction by radiation in several tumor cell lines, this was not the case in our study as neither cGAS nor STING were upregulated on mRNA level. However, we confirmed the activation of STING, but the induction of type I IFN (IFNβ) in tumor cells was minor compared to the induction of TNFα, which expression can also be activated by STING [21,29]. Therefore, every tumor cell type seems to have its unique response to radiation depending on the combination of activated DNA sensors.

The activation of DNA sensing pathways was more potent in RAW 264.7 macrophages as opposed to B16F10 tumor cells. A more pronounced upregulation followed the pattern of micronuclei and dsDNA accumulation in the cytosol after irradiation. Micronuclei clearly originate from the cell nucleus, however, dsDNA can be either nuclear or mitochondrial DNA, which is released upon irradiation-induced damage on DNA and nuclear or mitochondrial membranes. A negligible number of dsDNA spots were detected in the control nonirradiated macrophages cytosol. In contrast, more dsDNA was evident in control nonirradiated tumor cells. This indicates a possible altered response to cytosolic dsDNA in the tumor cells, as DNA can already be present in their cytosol. The presence of DNA in the cytosol of tumor cells was also demonstrated in previous studies primarily due to genomic instability [30,31]. This could explain the more potent response in RAW 264.7 macrophages, since these cells are the first line of defense against microorganisms and do not have a high baseline level of DNA in their cytosol, although they were derived from a tumor. The more potent response could also be attributed to higher radiosensibility of macrophages as toxic doses of radiation doses must be used in order to induce the DNA sensing pathways, as accumulation of micronuclei and dsDNA in the cytosol is due to the radiation-induced nuclear damage. For comparison of nontoxic doses, our data on 2 Gy irradiation can be considered, which demonstrated that 2 Gy are not toxic enough to induce the DNA sensing responses.

The activation of DNA sensors in response to high dose radiation was accompanied by the expression of different cytokines that can mediate distinct cellular responses. In our study, a cell type specific expression of cytokines was detected. B16F10 tumor cells expressed high levels of TNFα and moderate levels of IFNβ after irradiation. TNFα is an inflammatory cytokine which is involved in a diverse range of signaling events within cells, leading to necrosis or apoptosis [32]. Moreover, signaling through both expressed cytokines, TNFα and IFNβ can act synergistically to induce necroptosis [29]. Taken together, this signaling could reduce the survival of tumor cells. Therefore, activation of DNA sensing pathways in response to high dose radiation could be the mechanism that mediates RIBE, which we detected in reduced clonogenic survival of tumor cells after the transfer of cell culture medium from irradiated tumor cells to nonirradiated cells. The expression of the receptor mRNAs for both cytokines in tumor cells was confirmed. Therefore, it is possible that the released cytokines can act by both autocrine and also paracrine signaling, inducing tumor cell death in neighboring bystander cells.

The activation of DNA sensing pathways in macrophages was associated with increased expression of IFNβ and IL1β. IFNβ can mediate direct antitumor effects with its antiproliferative effect or induction of apoptosis [33]. Indeed, tumor cell death was observed when cell culture medium was transferred from high dose irradiated macrophages to nonirradiated tumor cells. 

In this study, it was also determined whether DNA released in response to high dose radiation could further enhance RIBE by entering bystander cells and inducing DNA sensing pathways. It was demonstrated that the entry of DNA is possible, since the EdU labeled DNA from cell-conditioned medium originating from irradiated tumor cells in a few fresh nonirradiated tumor cells was detected. However, the effect was rare and was not potent enough to induce the upregulation of the DNA sensors mRNAs. Nevertheless, this basic in vitro study may extend to the in vivo setting, so this mechanism may become significant in the case of solid tumors, which contain large numbers of distinct cell types and also larger concentrations of cfDNA have been described in studies in patients receiving conventional radiotherapy or SBRT [34,35]. However, cfDNA is probably not the only source of DNA, which was detected to enter into the bystander cells in our study. By our medium centrifugation protocol, we removed only the remaining cells and some part of cell debris with apoptotic bodies, microvesicles, exosomes, and other sorts of extracellular vesicles remaining in the cell medium. These entities can also contain DNA and their contribution to activating DNA sensing pathways should be considered in the future by ultracentrifugation of cell medium. Additionally, it has to be acknowledged that these extracellular vesicles can also contain other molecules such as reactive oxygen species (ROS), noncoding RNAs, and proteins, that can potentially impact the clonogenic survival of cells and therefore ultracentrifugation should also be utilized to fully distinguish the potential effects of different extracellular vesicles on clonogenic survival in terms of RIBE.

A limitation of our study is that it was an in vitro study, and did not fully address the heterogeneity of solid tumors. To fully address the heterogeneity of tumor microenvironment, other cells such as tumor-associated fibroblasts, endothelial cells, pericytes, dendritic cells, and others should be considered in the future. However, this in vitro setting on commercially available user-friendly cell lines allowed the complete differentiation of the effects on different cell types, which can now be confirmed and more comprehensively investigated on additional cell types or in in vivo setting in mouse tumor models or patient biopsies. Moreover, specific tumor types should be evaluated separately as based on this and other preclinical studies. With this analysis, we can anticipate that each tumor type would respond differently due to its histopathological characteristics, for example the fraction of distinct cell types or their spatial distribution. One important factor that should be taken into account in the future is the selection of doses and dose fractionation. Our study was limited to testing a single fraction high dose irradiation, which is typically used in SBRT, as we demonstrated that 2 Gy fraction that is used in conventional radiotherapy does not cause significant upregulation of DNA sensors and thus this proposed mechanism for off-target effects are not relevant for doses used in conventional radiotherapy. However, since it is known that radiation given as a single dose can induce different gene expression profiles when compared to radiation given in multiple fractions, which is also used in the clinical practice [36], in the future, fractionation with clinically relevant conventional and high dose irradiation doses should be evaluated in relevant human cell lines and tumor models. Furthermore, higher radiation doses or late time point, beyond the initial apoptotic response should be evaluated.

To summarize, this study demonstrates that high dose irradiation which is used in SBRT and SRS activates the DNA sensing pathways and that this is an important mechanism mediating the induction of stimulatory cytokines, which can mediate bystander and abscopal effects in solid tumors. The study demonstrates that the activation differs based on the cell type and is time- and radiation dose-dependent, as this effect was not demonstrated for doses used in conventional radiotherapy. Every cell type may have its unique response to irradiation depending on the combination of activated DNA sensors. Understanding the timing of the activation of these pathways and subsequent cytokine expression induced by different irradiation doses also in fractionated schemes could improve the schedules for combining immune checkpoint inhibitors or other immunotherapies with the radiation therapy in order to improve the therapy of cancer.

## 4. Materials and Methods

### 4.1. Experimental Design

This study was designed to investigate the involvement of different DNA sensing pathways in response to irradiation with two hypotheses: (1) multiple DNA sensing pathways are involved in the response to irradiation and (2) DNA sensing pathways are involved in the off-target effects of radiation (bystander effect or abscopal effect). Gene expression experiments and flow cytometry were performed to explore the changes in DNA sensors and cytokine expression. Immunofluorescence imaging was used to follow the activation of signaling proteins involved in DNA sensing by following their translocations through different cell compartments. The transfer of conditioned medium from irradiated to nonirradiated cells was the basis for the evaluation of RIBE. Each experiment was performed independently at least three times and contained biological or technical replicates where appropriate, as specified in figure legends; no outliers were excluded. Collection points were determined based on survival data; we limited the study to the early response of cells at 24 and 48 h after irradiation, prior to radiation-induced cell death in tumor cells. The study was designed as a basic in vitro study on two different cell lines (one tumor and one macrophage cell line) with the exploratory setting investigating the possible involvement of DNA sensing pathways in radiation-induced bystander effect. As an in vitro study, the study allowed us to fully distinguish the effects between different types of cells and to detect rare events such as the uptake of DNA from the cell-conditioned medium after irradiation were followed. The cells were randomly allocated to experimental groups and the investigators were not blinded to the experiments.

### 4.2. Cell Lines

B16F10 murine melanoma cell line (ATCC CRL-6475™) and RAW 264.7 murine macrophages (ATCC TIB-71™) were purchased from American Type Culture Collection (ATCC, Manassas, VA, USA). RAW 264.7 macrophages were purchased in 2019 and B16F10 cells were authenticated by STR profiling and interspecies contamination test in 2019 (IDEXX BioAnalytics, Ludwigsburg, Germany). The B16F10 cells and RAW 264.7 cells were cultured in Advanced Minimum Essential Medium (AMEM, Gibco, Thermo Fisher Scientific, Waltham, MA, USA) and Advanced Dulbecco’s Modified Eagle’s Medium (DMEM, Gibco), respectively. Cell media were supplemented with 5% fetal bovine serum (FBS, Gibco), GlutaMAX (100×, Gibco), 100 I.U./mL penicillin (Sandoz, Barleben, Germany), and 0.05 mg/mL gentamicin (Garamycin, Krka, Novo mesto, Slovenia). Cells were routinely subcultured twice a week with the use of 0.25% Trypsin-EDTA solution (B16F10) or cell scraping (RAW 264.7) for detachment and maintained in a 5% CO_2_ humidified incubator at 37 °C. The cells were routinely tested for mycoplasma infection by MycoAlertTM PLUS Mycoplasma Detection Kit (Lonza, Basel, Switzerland) and were mycoplasma free. Doubling times of cells were 16 h for B16F10 cells and 22 h for RAW 264.7, therefore, comparable number of divisions were anticipated after the irradiation.

### 4.3. Irradiation

Gulmay CP225 X-Ray Generator (Gulmay Medical Ltd., Byfleet, UK) with 0.55 mm copper and 1.8 mm aluminum filtering was used for irradiation of cells. Dose rate was 1.8 Gy/min and 2, 4, 6, or 8 Gy were delivered to the cells.

### 4.4. Determination of Cell Survival

A mixture of 1000 cells in 100 μL of cell culture medium was incubated in 5% CO_2_ humidified incubator at 37 °C for 2–3 h in the wells of a 96-well flat bottom plate. After cell attachment, plates were irradiated with 2, 4, 6, or 8 Gy then incubated for 24, 48, or 72 h. Cell survival was measured with a cell viability assay (PrestoBlue™ Cell Viability Reagent, Thermo Fisher Scientific), a cell permeable resazurin-based solution that quantifies the reducing power of living cells, for 1 h per manufacturer’s instructions. Fluorescence intensity was measured with a filter set at 535–554 nm (Excitation)/615–650 nm (Emmision) with a microplate reader (GENios, Tecan, Männedorf, Switzerland).

### 4.5. RNA Extraction, Reverse Transcription, and Quantitative Real-Time PCR (qRT-PCR)

For determination of DNA sensor and cytokine mRNA levels 24 and 48 h after irradiation, 5 × 10^5^ B16F10 cells in T75 flasks in 10 mL of cell culture medium and 5 × 10^5^ RAW 264.7 cells in T25 flasks in 5 mL of cell culture medium were plated and irradiated after 16 h of incubation. After 24 or 48 h, cell medium containing dead cells was removed and RNA was extracted using peqGOLD Total RNA Kit (VWR, West Chester, PA, USA) according to manufacturer’s instructions. RNA concentrations and purity were quantified by spectrophotometric measurements at 260 nm (Epoch, Biotek, Winooski, VT, USA) and by measuring the ratio of absorbance at A260 nm/280 nm, respectively. Afterwards, 500 ng of total RNA was reverse transcribed into cDNA using the SuperScript VILO cDNA Synthesis Kit (Invitrogen, Thermo Fisher Scientific) according to manufacturer’s instructions. After reverse transcription, 10x dilution of cDNA (12.5 ng) was used as a template in each 20 μL qRT-PCR reaction using custom primers (Integrated DNA Technologies, Coralville, IA, USA) (Table S1) and PowerUp™ SYBR™ Green Master Mix (Applied Biosystems, Thermo Fisher Scientific). The qRT-PCR reactions were performed, and the products analyzed on QuantStudio™ 3 Real-Time PCR System (Thermo Fisher Scientific). Relative quantification was performed by comparison to the housekeeping genes β-actin (*Ba*) and/or glyceraldehyde 3-phosphate dehydrogenase (*Gapdh*) method and normalized to nonirradiated cells (0 Gy group) using the ΔΔCt method. For determination of expression of cytokine receptors, relative quantification was performed by comparison to the *Gapdh* using ΔCt method. Non determined (N.D.) was annotated when Ct value was above 40th cycle.

### 4.6. Immunofluorescence

Immunofluorescence staining was used to detect the accumulation of dsDNA in the cytosol of cells and for detection of the translocation of STING, IRF3, IRF7, and NF-κb. A total of 2000 B16F10 cells or 5000 RAW 264.7 cells in 200 μL of cell culture media were seeded into each well of 12 Well Chamber (Ibidi, Gräfelfing, Germany) and incubated for 16 h. Chambers were then irradiated with 6 Gy with the exception of the control chamber. Chambers were incubated for 24 or 48 h and immunofluorescently stained. In order to recognize the staining pattern of active STING, transfection with G3-YSD (Invivogen, San Diego, CA, USA), a STING, as positive control was performed using Lipofectamine RNAiMAX (Thermo Fisher Scientific) in the control chamber 24 h prior to staining (Figure S3). Cells were fixed in 4% paraformaldehyde (PFA; Alfa Aesar, Thermo Fisher Scientific) for 15 min at 37 °C then the plasma membrane was stained with 1 μg/mL WGA AlexaFluor 647 conjugate solution (Thermo Fisher Scientific) in Hanks’ Balanced Salt Solution (HBSS, calcium, magnesium, no phenol red, Gibco, Thermo Fisher Scientific) for 10 min at room temperature (RT). Between these steps, the wells were washed twice with HBSS for 5 min at RT. Cells were then permeabilized with Tween 20 in phosphate-buffered saline (PBS) in 0.02% solution for dsDNA and STING staining for 15 min and in 0.5 % solution for IRF3, IRF7, and NF-κB staining for 20 min at RT. For dsDNA staining, an extremely mild detergent was used for permeabilization (0.02% Tween 20) that permeabilized only the plasma membrane and not the nuclear or mitochondrial membrane, which would result in a very bright signal that would mask the signal obtained from cytosolic DNA. After permeabilization, cells were washed twice with PBS for 5 min at RT. Nonspecific binding was blocked at RT for 1 h in blocking buffer (0.01% Tween 20, 5% donkey serum, 22.52 mg/mL glycine in PBS) for all staining protocols. A 0.01% solution of Tween 20 in the blocking buffer was used to prevent the nonspecific binding of primary antibodies. A mixture of primary antibodies in blocking solution was added and incubated overnight at 4 °C (see Table S2 for the complete list of antibodies, dilutions, and isotype controls). The next day, cells were washed three times with PBS for 5 min at RT and then incubated with the solution of Alexa Fluor 488 secondary antibodies in PBS for 1 h (Table S2). After the incubation, cells were washed twice with PBS for 5 min and nuclei were stained with 3 µg/mL of Hoechst solution (Hoechst 33342, Trihydrochloride, Trihydrate, Thermo Fisher Scientific) in PBS for 15 min at RT. Afterwards, cells were washed twice with PBS at RT for 5 min, the silicon wells removed, and the slides mounted in Prolong Gold Diamond Antifade Mount (Thermo Fisher Scientific) for 3 days at RT and then sealed with nail polish. Imaging was performed with an LSM 800 confocal microscope (Carl Zeiss, Oberkochen, Germany) with a 63× oil immersion objective (NA 1,4). Hoechst 33342, Alexa Fluor 488, and Alexa Fluor 647 were excited with lasers with excitation wavelengths of 405, 488, and 640 nm, respectively. The emitted light was collected sequentially with Gallium Arsenide Phosphide (GaAsP) detector via a variable dichroic and filters at the following wavelengths: 410–545 nm (Hoechst 33342), 488–545 nm (Alexa Fluor 488), and 645–700 nm (Alexa Fluor 647). The collected images or Z-stacks were then visualized in Imaris software (Bitplane, Zurich, Switzerland). The number of IRF3, IRF7, or NF-κB positive nuclei and the number of cells with peri-nuclear STING staining were quantified using cell counter plugin in Fiji software (ImageJ, NIH). The number of intracellular and extracellular dsDNA spots was quantified using Imaris software (Bitplane, Belfast, UK). Images were quantified by dividing the number of spots or positive nuclei with the number of cells on the image.

### 4.7. Flow Cytometry

Intracellular flow cytometry was used to evaluate intracellular cytokine expression 24 and 48 h after irradiation. A total of 500,000 B16F10 cells in T75 flasks in 10 mL of cell culture medium or 5 × 10^5^ RAW 264.7 macrophages in T25 flasks in 5 mL of cell culture medium were plated and incubated for 16 h. Afterwards, cells were irradiated with 6 Gy at two time points to achieve the 24 or 48 h incubation after irradiation. On the day of the measurements, eBioscience™ Protein Transport Inhibitor Cocktail (500×, Invitrogen, Thermo Fisher Scientific) was added into the cell medium and incubated for 2 h. After incubation with protein transport inhibitors, cells were detached from the surface and a suspension of 1 × 10^6^ cells was prepared in PBS. Cells were stained with eBioscience™ Fixable Viability Dye eFluor™ 780 (FVD, Thermo Fisher Scientific) for 30 min at 4 °C (1 μL per 1 mL of cell suspension). Cells were then fixed in eBioscience™ IC Fixation Buffer (Thermo Fisher Scientific) diluted 1:1 in PBS for 30 min at RT and permeabilized in 1× eBioscience™ Permeabilization Buffer (Thermo Fisher Scientific) during centrifugation (400 × g, 5 min). Afterwards, primary conjugated antibodies were added to stain the cytokines. In each B16F10 sample, 1 μL of Brilliant Violet 421™ anti-mouse TNF-α Antibody (MP6-XT22, Biolegend, San Diego, CA, USA) and 25 μL of reconstituted Mouse Interferon beta (IFNβ) AssayLite Antibody (APC Conjugate, ASSAYPRO, St. Charles, MO, USA) in 1 mL of PBS was added. In each RAW 264.7 sample, 10 μL of Mouse IL-1 beta/IL-1F2 Fluorescein-conjugated Antibody (R&D Systems, Minneapolis, ME, USA) and 25 μL of Mouse Interferon beta (IFNβ, AssayLite Antibody) was added. For fluorescence compensation, unstained control and single stained controls were used for each experiment. For unstained control and single stained for FVD (live/dead), a mixture of nonirradiated and irradiated cells was used. For TNFα, IFNβ, or IL1β single stained controls, 1 μL of antibodies were directly added to AbC™ Total Antibody Compensation Bead Kit (Thermo Fisher Scientific). A mixture of nonirradiated and irradiated cells was prepared for fluorescence minus one controls, which were used for appropriate gating of positive populations. The measurements were performed on at least 100,000 cells per sample using FACSCanto II flow cytometer (BD Biosciences, San Jose, CA, USA). Data were analyzed using FlowJo software.

### 4.8. Medium Transfer Method

A suspension of 5 × 10^5^ B16F10 cells or RAW 264.7 macrophages in 10 mL of cell culture medium was plated in T75 flasks. After incubation in 5% CO_2_ humidified incubator at 37 °C for 16 h, one group of cells was irradiated with 6 Gy and the other was not irradiated. After irradiation, cells were incubated again for 24 h to obtain cell-conditioned medium. The cell-conditioned medium from both groups was collected in a 50 mL centrifuge tube and centrifuged sequentially three times (470 × g, 5 min) in order to remove the remaining cells. The prepared cell-conditioned medium was then transferred to fresh B16F10 or RAW 264.7 cells and incubated for 24 h (Bystander 24 h–24 h group) or 48 h (Bystander 24 h–48 h group) in experiments evaluating the upregulation of DNA sensor and cytokine mRNAs as described previously.

### 4.9. Clonogenic Assay

The determination of the RIBE effect with the clonogenic assay was performed as follows. In each well of a 6-well plate, 50 cells in 3 mL of cell-conditioned medium were combined as described previously. The experimental group contained fresh B16F10 cells mixed with the cell-conditioned medium from the irradiated B16F10 cells or RAW 264.7 macrophages with 6 Gy (IR group). The control group contained fresh B16F10 cells mixed with the cell-conditioned medium from nonirradiated B16F10 cells or RAW 264.7 macrophages (Ctrl group). Cells were incubated in 5% CO_2_ humidified incubator at 37 °C for 6–8 days. Colonies were fixed and stained with crystal violet (Sigma-Aldrich, St. Louis, MO, USA) and counted. The colonies containing less than 50 cells were disregarded. Plating efficiency was calculated for each group as the ratio between counted colonies and the number of plated cells. Plating efficiency of the experimental group (IR group) was normalized to the control cells group (Ctrl group), representing surviving fraction.

### 4.10. Isolation, Analysis, and Quantification of DNA from Cell Culture Media

A suspension of 1 × 10^5^ B16F10 cells in 4 mL of cell culture medium was plated in T25 flask for isolation of DNA. After a 16 h of incubation, cells were irradiated with 6 Gy and additionally incubated for 24 or 48 h. Cell culture medium was then collected and centrifuged sequentially twice (470× *g*, 5 min) in order to remove the remaining cells. Afterwards, DNA was isolated from 3.5 mL of cell culture medium using a GenElute™ Plasma/Serum Cell-Free Circulating DNA Purification Midi Kit (Sigma Aldrich, Merck, Darmstadt, Germany). The DNA was eluted in 50 μL and 5 μL of solution was used to measure its concentration by the Qubit™ dsDNA HS Assay Kit (Thermo Fisher Scientific) and Qubit™ 4 Fluorometer (Thermo Fisher Scientific). The measured concentration of DNA after irradiation was normalized to the DNA concentration of control, nonirradiated cells. The DNA fragments (10 μL of eluted mixture) were also separated in 1.5% agarose gel in Tris-acetate-EDTA (TAE) buffer for 80 min at 100 V, cooled with ice. GeneRuler 1 kb DNA Ladder (Thermo Fisher Scientific) and Gel Loading Dye, Purple (6×), no SDS (New England Biolabs, Ipswich, MA, USA) were used for ladder and sample loading, respectively. After separation, the gel was stained with SYBR™ Gold Nucleic Acid Gel Stain (Thermo Fisher Scientific) for 30 min and fragments visualized using Gel documentation system (Vilber, Marne-la-Vallée, France).

### 4.11. Detection of Transfer of DNA with Incorporated EdU into Bystander Cells

The transfer of DNA with incorporated EdU from the cell-conditioned medium of irradiated B16F10 cells into the fresh nonirradiated B16F10 cells was followed by EdU immunofluorescent staining with Click-iT™ Plus EdU Cell Proliferation Kit for Imaging (Alexa Fluor™ 488 dye, Thermo Fisher Scientific). EdU (5-ethynyl-2′-deoxyuridine) is a nucleoside analog of thymidine, which is incorporated into DNA during active DNA synthesis. A suspension of 5 × 10^5^ B16F10 cells in 10 mL of cell culture medium was plated into two T75 flasks. After the attachment of the cells (2–3 h), half of the cell medium was replaced by the 10 μM solution of EdU and cell medium, which yielded a 5 μM final solution of EdU. After 16 h of incubation, the majority of cells divided and EdU was incorporated into their DNA, therefore the DNA, released into the cell medium was also expected to have EdU incorporated. After 16 h, the solution of EdU in cell medium was removed and the cells were extensively washed in order to remove the remaining EdU, which could interfere with the experiment. After washing, 10 mL of fresh cell medium was added. One flask was irradiated with 6 Gy; the control flask was not. After 24 h of incubation, 200 μL of cell-conditioned medium containing the DNA with incorporated EdU was transferred in each well of 12 Well Chamber (Ibidi), which contained 2000 fresh B16F10 cells. After 24 h incubation, cells were stained for EdU. Cells were fixed in 4% PFA for 15 min at RT and the plasma membrane was stained with 1 μg/mL WGA AlexaFluor 647 conjugate solution in HBSS for 10 min at RT. Between these steps, wells were washed twice with HBSS for 5 min at RT. Afterwards, cells were permeabilized with 0.02% solution of Tween 20 in PBS 20 min at RT then washed with the solution of 3% donkey serum in PBS. After this step, the Click-It Plus reaction cocktail containing Alexa Fluor 488 picolyl azide was prepared according to manufacturers’ instructions and added to the cells for 30 min at RT. Afterwards, cells were washed two times with PBS for 5 min and nuclei were stained with 3 µg/mL of Hoechst 33,342 solution in PBS for 15 min at RT. Cells were washed twice with PBS, the silicon wells were removed, and the slides were mounted in Prolong Gold Diamond Antifade Mount for 3 days at RT and then sealed with nail polish. Imaging was performed as described in immunofluorescence method section. Images were quantified by counting the number of EdU spots in each image and dividing it with the number of cells.

### 4.12. Statistical Analysis

All values in this study represent mean ± standard error of the mean (SE) unless otherwise stated. The data were first tested for normality of distribution with the Shapiro–Wilk test. Comparison between two groups was performed using unpaired two-tailed Student’s *t*-test. The comparison of means of more than two groups was statistically evaluated by one-way ANOVA followed by a Dunnett’s or Tukey’s multiple comparisons test as reported in each figure legend. A *p*-value of <0.05 was considered to be statistically significant. A sample size (*n*) for each experiment represents biological replicates unless otherwise stated in the figure legends. No statistical method was used to predetermine sample size. For statistical analysis and preparation of graphs GraphPad Prism 8 (La Jolla, CA, USA) was used.

## 5. Conclusions

We demonstrate that high dose irradiation which is used in SBRT and SRS activates the DNA sensing pathways and that this is an important mechanism mediating the induction of stimulatory cytokines, which can mediate the off-target bystander and abscopal effects in solid tumors. We propose that the activation differs based on the cell type and is time- and radiation dose-dependent, as this effect was not demonstrated for the doses used in conventional radiotherapy. Every cell type may have its unique response to irradiation depending on the combination of activated DNA sensors. Understanding the timing of the activation of these pathways and subsequent cytokine expression induced by different irradiation doses also in fractionated schemes could improve the treatment schemes for combining immune checkpoint inhibitors or other immunotherapies with the radiation therapy in order to improve the therapy of cancer.

## Supplementary Materials

The following are available online at https://www.mdpi.com/2072-6694/12/11/3365/s1, Figure S1. Bar graphs presenting statistically significant data from heat maps in Figure 1, Figure S2: Proposed scheme of cellular responses to radiation, Figure S3. Identification of positive immunofluorescence signal of STING activation using cGAS agonist G3- YSD in B16F10 cells, Figure S4: Original image of AGE gel, Table S1. List of qRT-PCR primers, Table S2. List of antibodies used for immunofluorescence.
