# Supplementary material for: Radiation Induced Upregulation of DNA Sensing Pathways is Cell-Type Dependent and Can Mediate the Off-Target Effects
Source: Cancers (Basel). 2020 Nov 13;12(11):3365. doi: 10.3390/cancers12113365 (PMC7696780; doi:10.3390/cancers12113365)
Supplement: Supplementary file 1 [file cancers-12-03365-s001.zip › cancers-971583-supplementary.docx]

Supplementary Materials: Radiation Induced Upregulation of DNA Sensing Pathways is Cell-Type Dependent and Can Mediate the Off-Target Effects

Tanja Jesenko, Masa Bosnjak, Bostjan Markelc, Gregor Sersa, Katarina Znidar, Loree Heller and Maja Cemazar


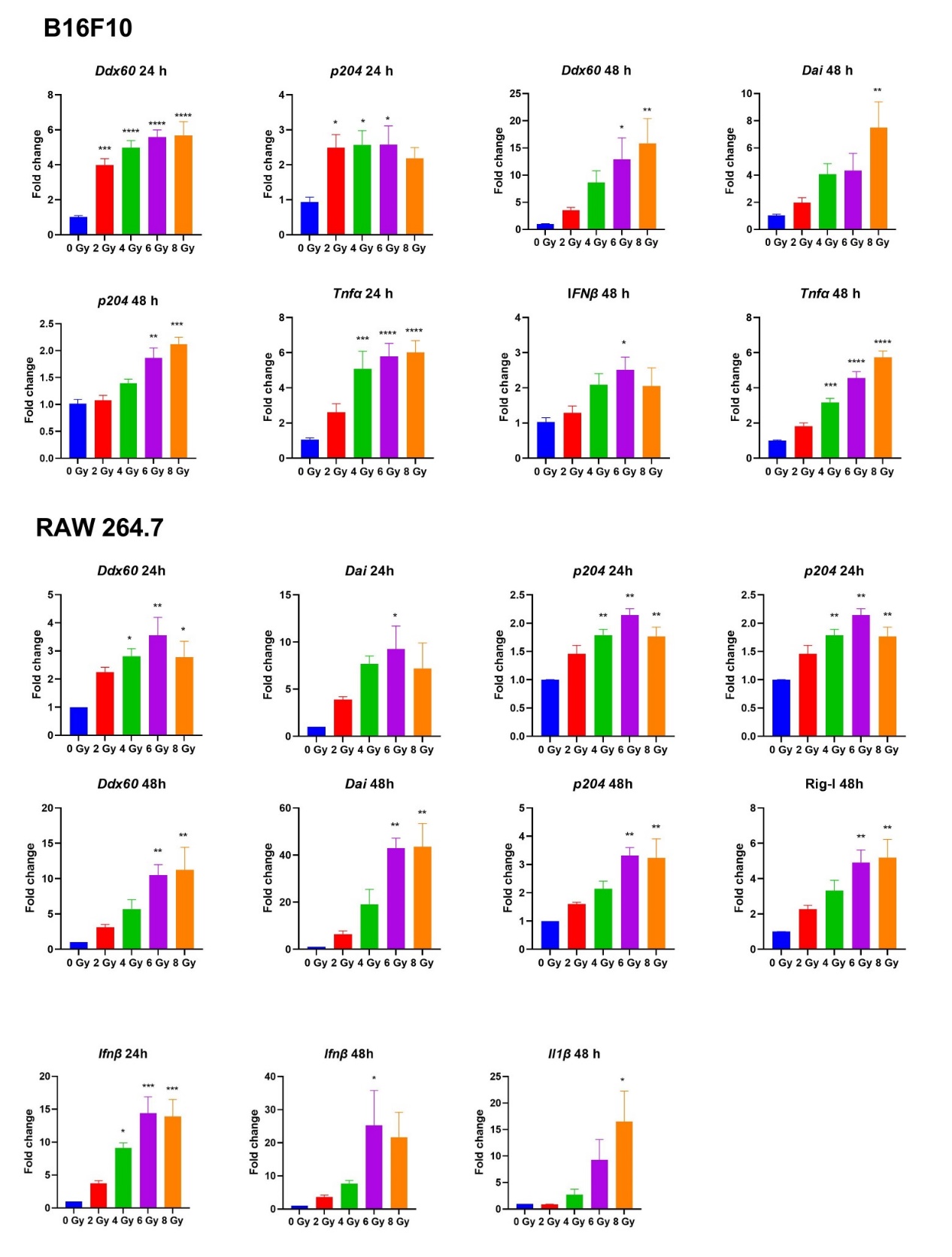


**Figure S1.** Bar graphs presenting statistically significant data from heat maps in Figure 1. * *p* < 0.05, ** *p* < 0.01, *** *p* < 0.001, **** *p* < 0.0001 vs. 0 Gy.

Based on our expression experiments and imaging studies, we propose possible schemes of cellular responses to high dose irradiation in terms of DNA sensing pathways in B16F10 tumor cells and RAW 264.7 macrophages (Figure S2). In both cell types, radiation damage caused the accumulation of DNA in the cytosol. In tumors cells, cytosolic DNA upregulated three DNA sensors, *Ddx60*, *Dai* and *p204*. The signals were then transferred to IRF3 and NF-κB transcription factors directly or through STING, which resulted in the transcription and subsequent expression of TNFα and IFNβ. In tumor cells, DNA was also demonstrated to exit the cells in the form of cfDNA, which was able to enter the neighboring (bystander) cells. In macrophages, cytosolic DNA upregulated *Ddx60,* *Dai* and *p204* and additionally *Rig-I*. However, the signal was transferred to IRF3, IRF7 and NF-κB transcription factors directly or through STING and resulted in transcription and subsequent expression of IFNβ and IL1β. Therefore, though similar DNA sensors were upregulated, the responses of cells in terms of cytokine expression were different, indicating that the response differs between the cell types.


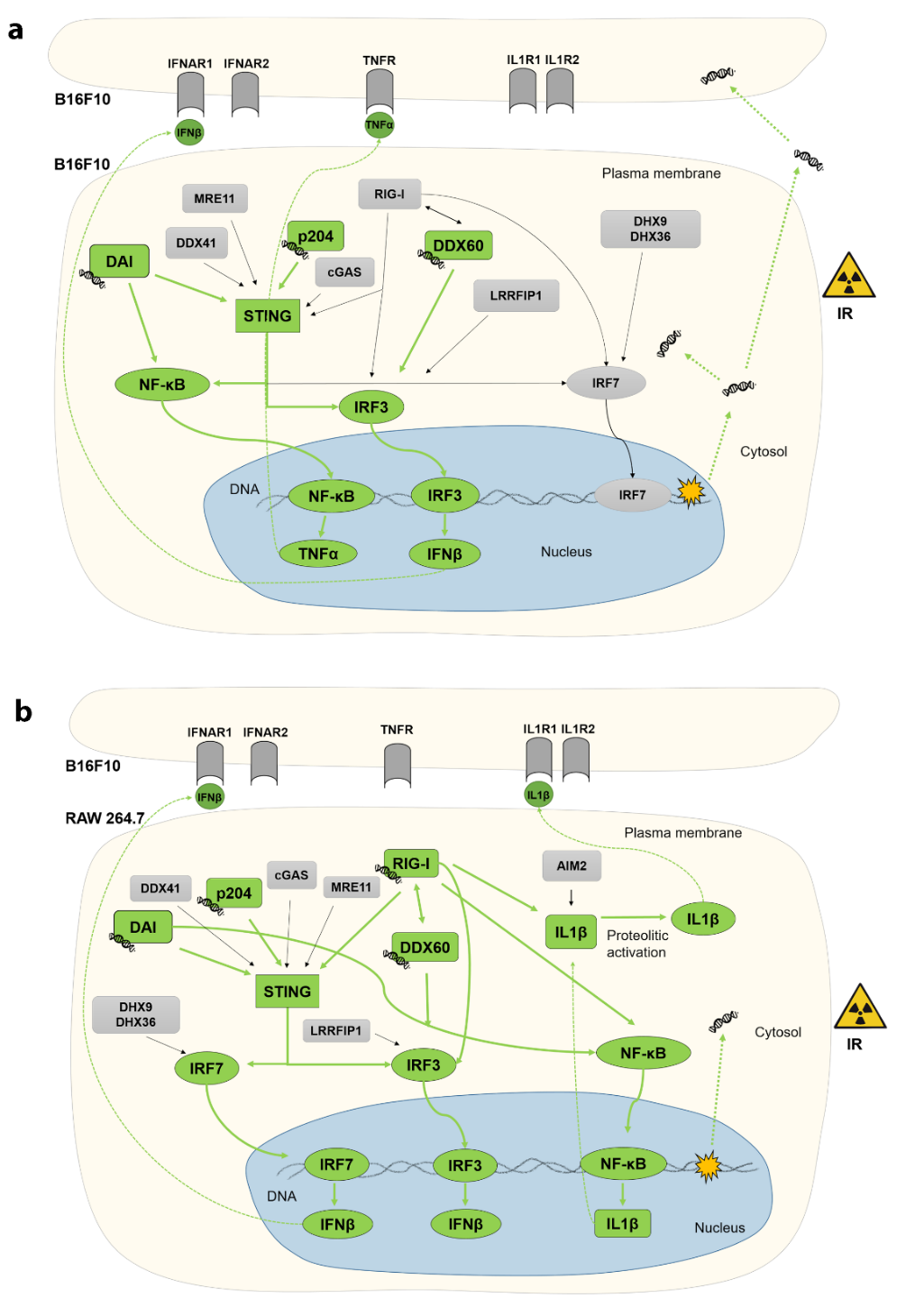


**Figure S2.** Proposed scheme of cellular responses to radiation in terms of DNA sensing pathways in (**a**) B16F10 tumor cells and (**b**) RAW 264.7 macrophages. The proposed scheme is based on our expression experiments and image studies. In both cell types, radiation damage caused the accumulation of DNA in the cytosol. The figure represents only the molecules, which were evaluated. Molecules denoted by green color were upregulated or activated in response to irradiation and the molecules in gray color were expressed in the cells, but were not upregulated or activated. IR- irradiation; DDX60 (DExD/H-Box helicase 6), DAI (DNA-dependent activator of IFN-regulatory factors), p204 (Interferon-inducible p204 protein), DDX41 (DExD/H-Box helicase 41), MRE11 (MRE11 Homolog, Double strand break repair nuclease), cGAS (cyclic GMP-AMP synthase), RIG-I (retinoic acid-inducible gene I), LRRFIP1 (LRR Binding FLII Interacting Protein 1), DHX9 (DExH-Box helicase 9), DHX36 (DExH-Box helicase 36), AIM2 (Absent in melanoma 2), STING (Stimulator of interferon genes), IRF3 (Interferon regulatory factor 3), IRF7 (Interferon regulatory factor) 7, NF-κB (Nuclear Factor kappa-light-chain-enhancer of activated B cells), TNFα (Tumor necrosis factor alpha), IFNβ (Interferon beta), IL1β (Interleukin 1 Beta), IFNAR 1 and 2 (Interferon-alpha/beta receptor.I and II), TNFR (Tumor necrosis factor receptor), ILIR1 and IL1R2 (Interleukin-1 receptor 1 and 2).


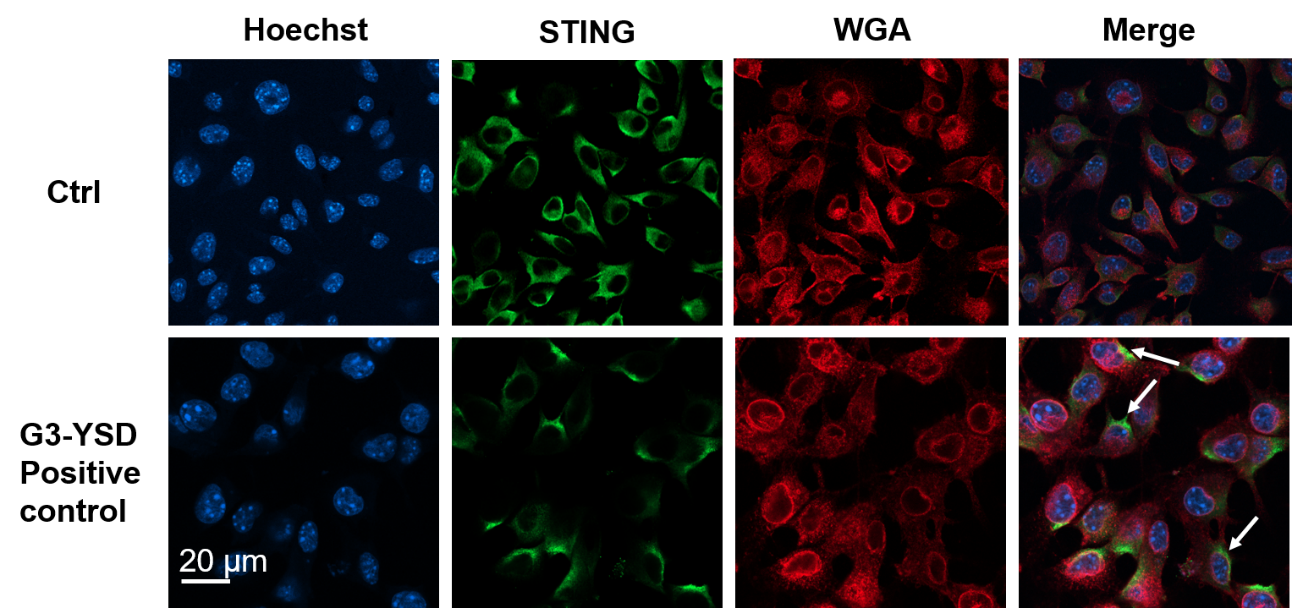


**Figure S3.** Identification of positive immunofluorescence signal of STING activation using cGAS agonist G3-YSD in B16F10 cells. Arrows indicate positive signal-translocation to peri-nuclear structures. Scale bar = 20 μm.


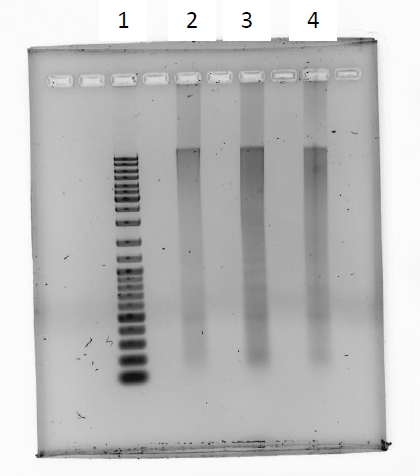


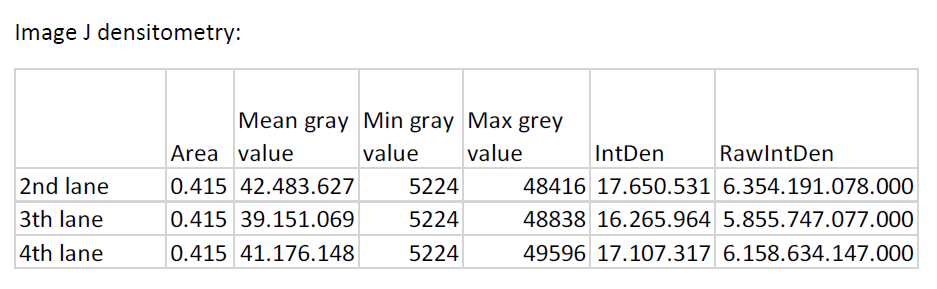


**Figure S4.** Original image of AGE gel. Left to right: 1st lane: GeneRuler 1 kb DNA Ladder (Thermo Fisher Scientific), 2nd lane: Ctrl, 3th lane: IR 24 h, 4th lane: IR 48 h.

**Table S1.** List of qRT-PCR primers.

| **Target** | **Sequence (5′-3′)** |
| --- | --- |
| *Ba_F* | GAAGTGTGACGTTGACATCC |
| *Ba_R* | ACTCATCGTACTCCTGCTTG |
| *Gapdh_F* | TTCACCACCATGGAGAAGGC |
| *Gapdh_R* | GGCATGGACTGTGGTCATGA |
| *cGAS_F* | GTGAGGACCAATCTAAGACGAG |
| *cGAS_R* | AGCATGTTTTCTCTATCCCGTG |
| *Dai_F* | TGCTTTCTAGAGGACGCCACCATT |
| *Dai_R* | TGGCTTCAGAGCTTGTACCTGTGT |
| *Sting_F* | GTCCTCTATAAGTCCCTAAGCA |
| *Sting_R* | AAGATCAACCGCAAGTACCC |
| *Ddx41_F* | AGTGCTCATGGACCTCAAAG |
| *Ddx41_R* | CTCCACAGAAGGCACAGC |
| *Ddx60_F* | ACTGGAACACTCGCTTTGG |
| *Ddx60_R* | GAAGTAGACATCACCCAACAGG |
| *Dhx9_F* | AAACTCCCCATTGAACCTCG |
| *Dhx9_R* | TGTATCCCAGGCGTTTTCC |
| *Dhx36_F* | CCCAAAGTTGCTAAAATCCGAC |
| *Dhx36_R* | CCAGTTGTAGTGGAAGTCTGTC |
| *Lrrfip1_F* | CCAGTTTGCCGAAGTGAAAG |
| *Lrrfip1_R* | CTCTCCGTTGGTGGCTATTT |
| *p204_F* | CCAGTCACCAATACTCCACAG |
| *p204_R* | GAGCACCATCACTGTCAGG |
| *Rig-I_F* | GAGCCAGCGGAGATAACAATA |
| *Rig-I_R* | CCCACGTACTCATAGAGAATGAC |
| *Mre11_F* | GAGAAAGGAAGCACAAAACTC |
| *Mre11_R* | TCACAAACATCCGATAGAGCC |
| *Aim2_F* | CCACCCGCAGTGACAATGACTTTA |
| *Aim2_R* | GCTTTCAGCACCGTGACAACAAGT |
| *Ifnβ1_F* | TGGCCATCCAAGAGATGCTCCAGA |
| *Ifnβ1_R* | AGAAACACTGTCTGCTGGTGGAGT |
| *Il1β_F* | AGTTGACGGACCCCAAAAGA |
| *Il1β_R* | TGCTGCTGCGAGATTTGAAG |
| *Tnfα_F* | CCCTCCAGAAAAGACACCATG |
| *Tnfα_R* | GTCTGGGCCATAGAACTGATG |
| *Ifnar1_F* | TCTCTGTCATGGTCCTTTATGC |
| *Ifnar1_R* | CTCAGCCGTCAGAAGTACAAG |
| *Ifnar2_F* | GTGACAGATAAGTGGTTGGAGG |
| *Ifnar2_R* | ACGATCTCAAATTCTGGCGG |
| *Tnfr_F* | CCCTCCAGAAAAGACACCATG |
| *Tnfr_R* | GTCTGGGCCATAGAACTGATG |
| *Il1r1_F* | AATGAGACGATCGAAGCTGAC |
| *Il1r1_R* | TTCTGATCCATTCCACTTCCAG |
| *Il1r2_F* | TGCTTTCACCACTCCAACAG |
| *Il1r2_R* | CCTTCCAGCCTCAATTCAGAT |

**Table S2.** List of antibodies used for immunofluorescence.

| **Target** | **Supplier** | **Cat. No.** | **Dilution** |
| --- | --- | --- | --- |
| dsDNA | Abcam | ab27156 | 1:1000 |
| STING | Novus Biologicals | NBP2-24683 | 1:200 |
| IRF3 | Santa Cruz Biotechnology | sc-9082 | 1:50 |
| IRF7 | Thermo Fisher Scientific | PA520280 | 1:100 |
| NF-κB | Abcam | ab140751 | 1:100 |
| Donkey Anti-Rabbit IgG Antibody (Alexa Fluor^®^ 488) | Jackson Immunoresearch | 711-545-152 | 1:500 |
| Donkey Anti-Chicken IgG Antibody (Alexa Fluor^®^ 488) | Jackson Immunoresearch | 703-545-155 | 1:500 |

**Publisher’s Note:** MDPI stays neutral with regard to jurisdictional claims in published maps and institutional affiliations.

| 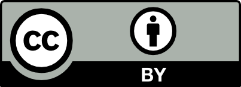 | © 2020 by the authors. Licensee MDPI, Basel, Switzerland. This article is an open access article distributed under the terms and conditions of the Creative Commons Attribution (CC BY) license (http://creativecommons.org/licenses/by/4.0/). |
| --- | --- |
